# An Exploration of the Metal Dependent Selectivity of a Metalloporphyrins Coated Quartz Microbalances Array

**DOI:** 10.3390/s16101640

**Published:** 2016-10-04

**Authors:** Alexandro Catini, Raj Kumar, Rosamaria Capuano, Eugenio Martinelli, Roberto Paolesse, Corrado di Natale

**Affiliations:** 1 Department of Electronic Engineering, University of Rome Tor Vergata, Via Politecnico 1, 00133 Roma, Italy; catini@ing.uniroma2.it (A.C.); rajkumar.lmd@gmail.com (R.K.); capuano@ing.uniroma2.it (R.C.); martinelli@ing.uniroma2.it (E.M.); 2 Department of Chemical Science and Technology, University of Rome Tor Vergata, Via della Ricerca Scientifica, 00133 Roma, Italy; roberto.paolesse@uniroma2.it

**Keywords:** electronic nose, metalloporphyrins, quartz microbalance

## Abstract

Several studies in the last two decades have demonstrated that metalloporphyrins coated quartz microbalances can be fruitfully used in many diverse applications, spanning from medical diagnosis to environmental control. This large versatility is due to the combination of the flexibility of metalloporphyrins molecular design with the independence of the quartz microbalance signal from the interaction mechanisms. The nature of the metal atom in the metalloporphyrins is often indicated as one of the most effective tools to design differently selective sensors. However, the properties of sensors are also strongly affected by the characteristics of the transducer. In this paper, the role of the metal atom is investigated studying the response, to various volatile compounds, of six quartz microbalance sensors that are based on the same porphyrin but with different metals. Results show that, since quartz microbalances (QMB) transducers can sense all the interactions between porphyrin and volatile compounds, the metal ion does not completely determine the sensor behaviour. Rather, the sensors based on the same molecular ring but with different metal ions show a non negligible common behaviour. However, even if limited, the different metals still confer peculiar properties to the sensors and might drive the sensor array identification of the pool of tested volatile compounds.

## 1. Introduction

Porphyrins chemistry offers excellent opportunities for gas sensors development [1]. The porphyrin macrocycle may harbor a number of interaction sites ruled by different interaction mechanisms. The macrocycle itself, the peripheral compounds, and the metal atom complexed at the core of the macrocycle drive the overall sensing characteristics.

The main stream in sensors development is directed towards the preparation of selective sensors; typically, stronger interactions tend to be more selective. Considering porphyrins, this approach privileges the coordination interactions between analytes and the metal ion of the metalloporphyrin complex. However to make a sensor, the sensitive material has to be complemented by a device that actually measure the physical quantity affected by the interaction. Then, for a selective sensor, the basic device only has to measure the consequences of the selective interaction. Excellent examples of selective porphyrins sensors are provided by oxygen sensors coupled with an optical read-out measuring the fluorescence changes [2] .

On the other hand, a different approach to sensors development considers the whole range of interactions and the sensors as elements of arrays. In this approach, the selectivity is a property of the array, and it is achieved by the combined effort of all the sensors. A similar situation occurs in olfaction where a limited number of receptors (about 300 in humans) can detect a huge number of different mixtures of volatile compounds [3] . Since olfactory receptors are partially selective, it is the pattern of responses that gives rise to the so-called combinatorial selectivity [4].

Mass sensors are a natural choice for such a sensor array. Indeed, regardless of the strength and the nature of the interaction, each bounded molecule contributes to an increase in the mass of the sensing layer. Several mass sensors are currently available. Usually, they are resonant structures made from piezoelectric crystals or micro-cantilevers. The first option is the most used, and different resonators can be fabricated with piezoelectric materials; these range from quartz microbalances to surface acoustic wave devices [5]. A fundamental parameter that defines the sensitivity of the device is the frequency of the oscillations, which spans a wide range from the few MHz of quartz microbalances up to GHz of devices made in GaN or AlN [6]. High frequency devices provide a great sensitivity, but at the same time they require more complex electronic circuits that also result in more noisy signals.

Quartz microbalances (QMBs) are the most basic mass sensors [7]. They are the same quartz crystals that are extensively used in electronics to drive accurate and stable clock signals. The frequency of the fundamental resonance mode of QMBs does not exceed a few tens of MHz. QMBs have been used for chemical sensors and biosensors for decades [8], and they have been frequently functionalized with porphyrins [9,10]. The capabilities of sensor arrays based on porphyrins have been demonstrated in very different applications, such as lung cancer detection from breath analysis [11,12] the contamination of foods [13], and the air quality control in spacecrafts [14].

In this paper, we investigated the role of metal ion in metalloporphyrins based QMB gas sensors. A homologous series of Tetraphenylporphyrins functionalized with six different metals have been exposed to a pool of volatile organic compounds (VOCs) representative of different chemical families. Results show that the behaviour of the sensors is characterized by a common mode, that is over impressed, and within which the peculiar property of each metalloporphyrins is found. This common mode is supposed to originate from all those molecular interactions that are not affected by the metal ion. Proper data treatment allows for the complete identification of the compounds regardless of the concentration. Each sensor contributes to the array, and peculiar association between sensors and VOCs can be observed. These results shed light on the positive results achieved by porphyrins based QMB arrays and also provide a methodology for sensor array design.

## 2. Experimental Section

Metal complexes of 5,10,15,20-Tetraphenylporphyrin (TPP) with copper (CuTPP), cobalt(II) (CoTPP), zinc (ZnTPP), manganese chloride (MnTPPCl), Iron chloride (FeTPPCl), and tin dichloride (SnTPPCl2) have been prepared following methods in the literature [15].

QMBs had a fundamental resonance frequency of 20 MHz, corresponding to a mass resolution in the order of a few nanograms. Thin films of sensing materials were deposited by a spray-coating on both sides of the quartz disks, from $10^{-3}$ M solution of porphyrins in CHCl${}_{3}$. For each sensor, the total coating resulted in a frequency shift of about 60 KHz. The sensors were housed in a stainless steel measurement chamber that had a volume of 10 mL. Sensors were connected to oscillator circuits. Frequencies were measured by means of an integrated frequency counter and then stored on a computer. The difference of the sensors signals in reference nitrogen gas and in VOCs enriched nitrogen gas was considered as the sensor response.

Sensors were calibrated measuring their response to a series of compounds representative of different chemical families, such as propanoic acid, ethanol, triethylamine, hexane, toluene, and dimethysulfide.

Vapors were generated by bubbling an N${}_{2}$ stream into a liquid sample of the compounds and diluted with nitrogen gas. The dilution rate was controlled by a computer-driven 4 channels mass-flow controller (MKS). The concentration of VOCs was calculated by Antoine’s law using the parameters listed in the NIST database [16].

## 3. Results and Discussion

The sensing properties of porphyrins functionalized QMBs have been characterized exposing the sensors to increasing concentrations of six VOCs. Each of these compounds can interact with different Van der Waals forces and hydrogen bonds. The intensity of the interactions that these VOCs can establish can be modelled by the Linear Sorption-Energy Relationship (LSER) [17]. The interactions considered in LSER are: polarizability (*R*), dipolarity (*π*), hydrogen bond acidity ($\alpha^{H}$) and basicity ($\beta^{H}$), and finally the solubility term ($LogL^{16}$), related to dispersion interactions.

Except for triethylamine and toluene, which lack the hydrogen bond acceptor and the hydrogen bond donor terms respectively, and hexane, for which only dispersion interactions can occur, all the other VOCs interact with all of the five mechanisms but with different relative strengths. The LSER coefficients of the tested VOCs are shown in Table 1.

**Figure 1:**
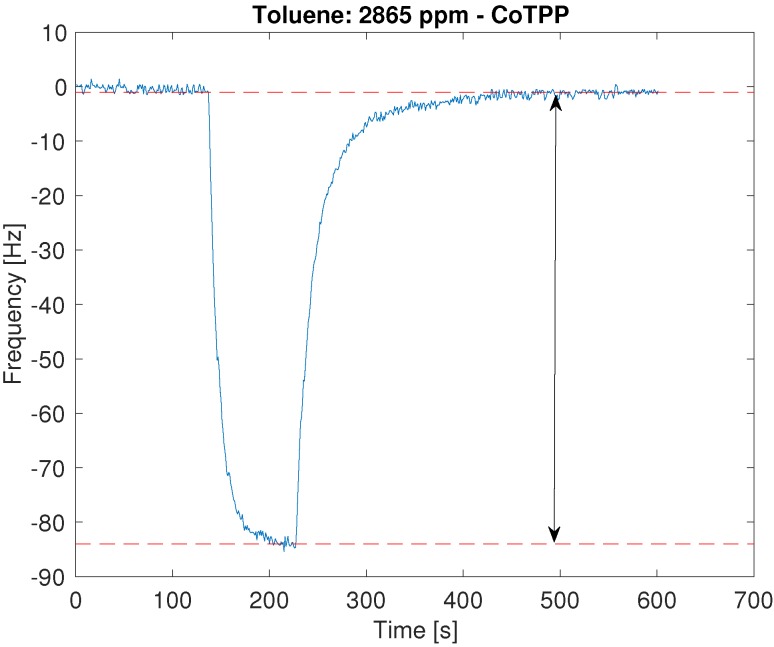
Time progression of the frequency shift of the sensor functionalized with CoTPP. The sensor is constantly kept in a nitrogen flow, from *t* = 130 s to *t* = 230 s 2865 ppm of toluene are added to nitrogen flow. After removal of the toluene, the sensor signal recovers the baseline. The line with two arrows indicates the sensor response used in the successive analysis.

All sensors show a reversible interaction with the VOCs and a signal proportional to the concentration. Figure 1 shows the typical sensor signal during one exposure; the sensor response was evaluated as the frequency shift between the resonance frequency measured immediately before the exposure and at the end of the exposure.

**Figure 2:**
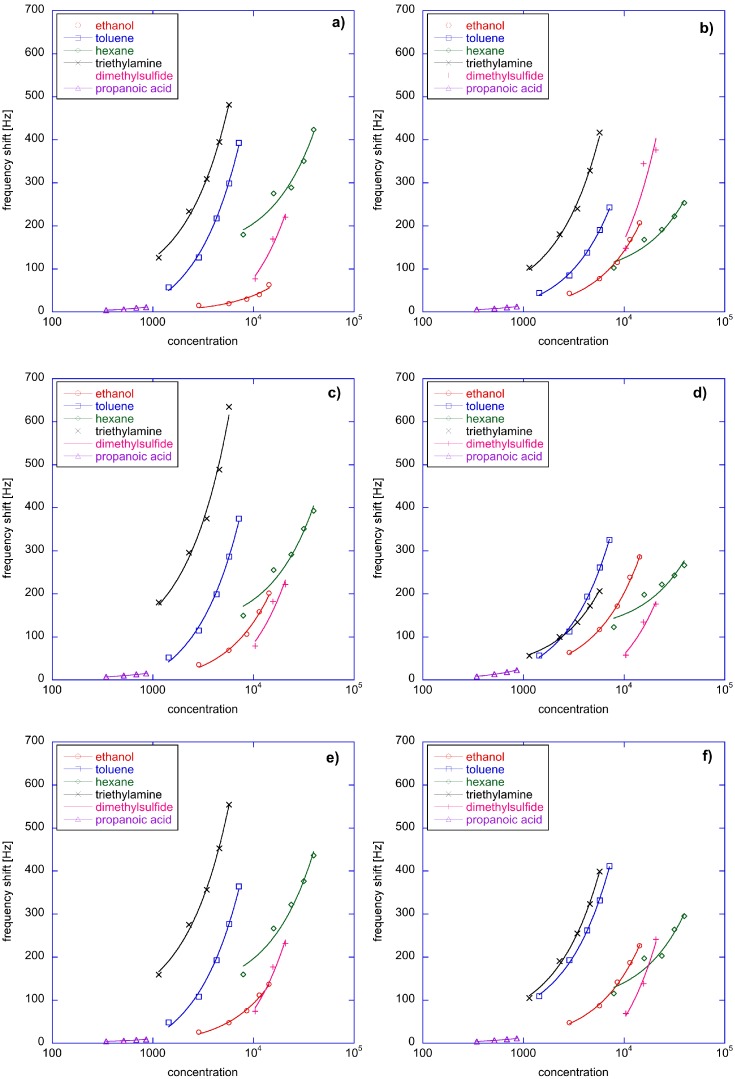
Response curves of each sensor vs. the concentration of test VOCs: (**a**) CuTPP; (**b**) CoTPP, (**c**) ZnTPP; (**d**) MnTPPCl; (**e**) FeTPPCl; (**f**) SnTPPCl2. In order to accommodate the wide range of concentration, the x axis is plotted in logarithmic scale. Linear fits, drawn as a continuous line, appears curved.

Figure 2 shows the response curve of each sensor towards the six compounds. In order to accommodate the different concentration ranges, the concentration axis is represented in a logarithmic scale. All sensor responses are characterized by a similar behaviour but with important differences. These differences appear subtle when response curves are compared, but, as it will be seen later, they become of great importance when the sensors are gathered into an array.

To better appraise the different behaviour of the sensors, the sensitivity has been evaluated. The sensitivity of the sensor is defined as the derivative of the sensor response with respect to the concentration [18], then in case of a linear relationship the sensitivity is independent from the concentration. In Figure 1, the linear fits of sensor response towards concentration are also plotted.

**Figure 3:**
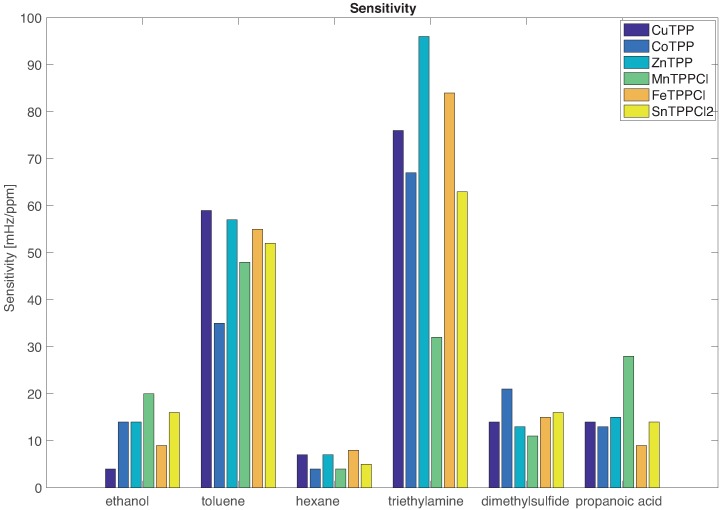
Sensors sensitivity respect to each VOC.

The sensors sensitivities are shown in Figure 3. The comparison between the sensitivities confirms that sensors are characterized by a common mode evidenced by the fact that the sensitivity to the VOCs proceeds in the same order for all sensors.

The individual character of the sensors emerges in the subtle differences that are imposed on the common mode. For instance, all sensors are more sensitive to triethylamine except MnTPPCl for which the largest sensitivity is towards toluene. The largest sensitivity towards ethanol and propanoic acid is achieved by MnTPPCl while ZnTPP is more sensitive to the largest electron donor (triethylamine).

The individual behaviour of sensors shows the intrinsic non selectivity of porphyrins coated QMBs. This character has to be ascribed to both the porphyrins and the QMBs. Indeed, the rich chemistry of porphyrins endow them with a number of different binding sites enabled by the different mechanisms. In this paper we study a homologous series of tetraphenylporphyrin which differs only by the metal ion complexed at the centre of the macrocycle. Then it is rather obvious to expect that a large part of chemistry is shared by these molecules. On the other hand, the metal ion strongly influences several porphyrin characteristics such as the solid-state arrangement. Eventually, the metal itself is a site for coordination binding of volatile compounds.

It is also worth noting that, in some of these porphyrins, the metal ion is complemented by chloride ions. The chloride ions are the counterions needed to balance the charge of the coordinated metal in order to keep the electroneutrality of the final complex. These ions can have different influences in the binding mechanism of the metalloporphyrins: for the axial coordination of the target VOC, in some cases, they should be displaced (such as, for example, in the case of SnTPPCl2). In addition, they could also influence the structure of the solid film of the metalloporphyrin, since they can form an obstacle for the π−π packing of the macrocycles, resulting in a more porous film.

On the other hand, QMB is a non selective transducer. Namely, all the adsorptions occurring between the sensing layer and the VOCs contribute to change the mass kept in movement by the piezoelectric crystal. It is interesting to observe that the situation may be very different when other transduction mechanisms are considered. For instance, Bohner et al. [19] showed that the sensitivity of phthalocyanine based chemoresistors is almost totally driven by the Lewis acid-bases interactions with a noteworthy exception of trimethylamine. Indeed, as seen in Table 1, triethylamine, besides being a strong donor, is also endowed with a large dispersion coefficient. Then the response of a QMB to triethylamine might be due to the combination of these two interactions. Furthermore, coordination dominates the sensitivity of the optical changes of porphyrins measured with spectrophotometers [20] or digital colorimeters [21].

QMB sensors are fully exploited when they are used as elements of sensor arrays and when multivariate data analysis is used to process the sensors data. For this scope, the data shown in Figure 2 are rearranged in a matrix where the rows are the measured samples and the columns are the sensors.

**Figure 4:**
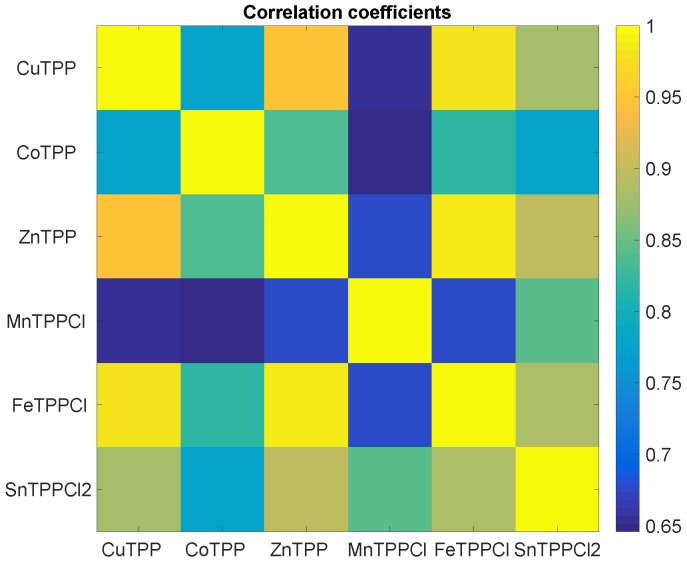
Map of linear correlations of sensors data. The magnitude of correlation is given in a color scale.

It is straightforward to understand that the common behaviour of sensors evidenced in Figure 2 and Figure 3 means that sensors data are correlated. Figure 4 shows the map of the linear correlation of the sensors. Correlation coefficients are greater than 0.65, and MnTPPCl is the sensor less correlated with the others.

The geometrical meaning of the above introduced data matrix is that each data point is a vector in a multidimensional space where the coordinates are defined by the sensors. In practice, given six sensors, the array data are points in a 6-dimensional space, where each sensor is a base vector of the space.

The simplest visualization of multivariate data sets is offered by the Principal Component Analysis (PCA) [22]. PCA aims at representing the data points in an orthogonal basis of uncorrelated variables (the principal components) that are obtained as a linear combination of the original base vectors (the sensors). In this way, the total variance of the data set is simply the sum of the variance carried by each principal component. As a consequence, a hierarchy among the principal components is established, and the representation can be limited to the components carrying most of the total variance. Furthermore, in PCA the components with large variance express the common modes of the sensor array.

**Figure 5:**
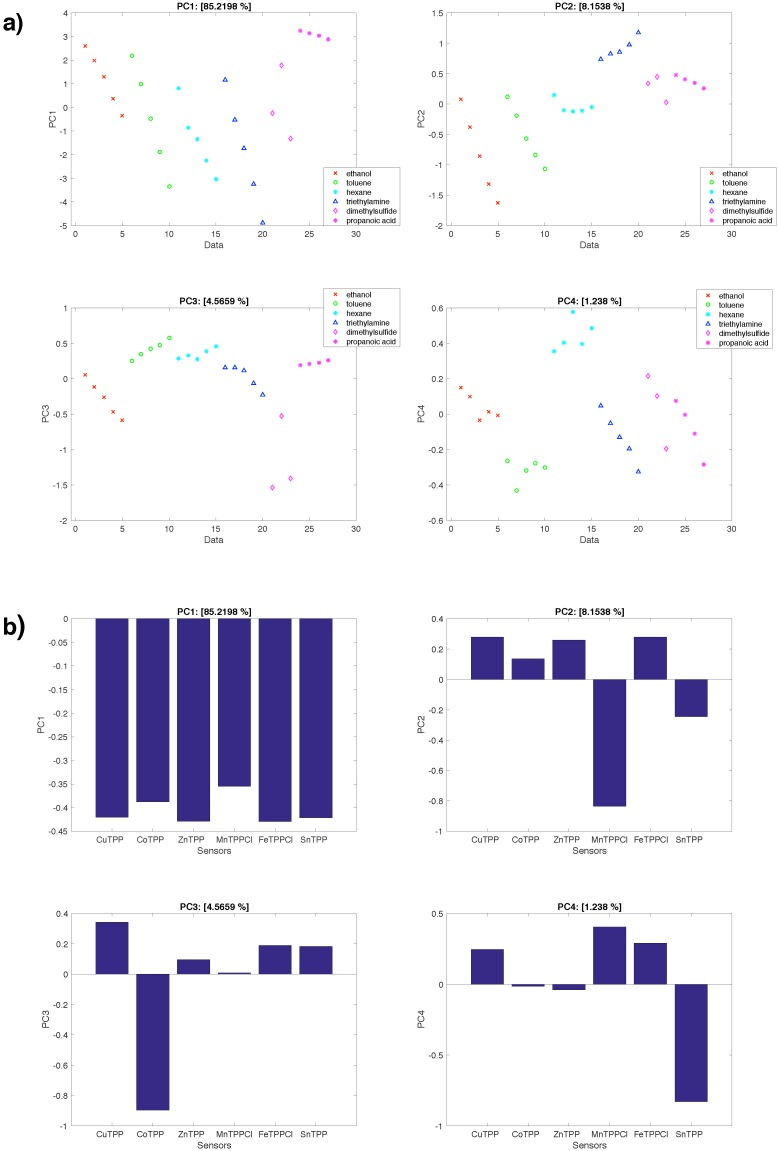
Results of the PCA of the sensors data. (**a**) Scores of the first four principal components; (**b**) Loadings of the first four principal components.

Figure 5 shows the scores (coordinates of the data points in the new base) and the loadings (contributions of each sensor to the principal components) of the sensors dataset. The matrix has been autoscaled. In other words, each column of the data matrix (each sensor) has been scaled to zero mean and unitary variance. It is important to remember that this normalization removes any influence due to different ranges of sensor responses. It has to be noted, that, in the case of functionalized QMBs, the sensor response is to a first approximation proportional to the amount of deposited sensing material. Then, such a normalization also reduces the influence of a non homogenous sensors deposition. Results are limited to the first four principal components for a total of more than 99% of total variance.

The first principal component carries 85% of the total variance. The scores of the different VOCs (Figure 5a) are not separated but they progress according to the concentration. In practice, PC${}_{1}$ captures most of the quantitative information (the amount of concentration) of each measure but little of the qualitative information (the nature of the VOC). This can also be understood considering that all the sensors equally contribute the loadings of the first principal component (Figure 5b).

A different situation is found in the other principal components. In PC${}_{2}$ (8% of total variance) a separation between triethylamine (positive score) and ethanol and toluene (negative scores) is observed. In terms of loadings, this separation is due to MnTPPCl and SnTPPCl2 in the negative portion and CuTPP, ZnTPP and FeTPPCl in the positive direction. Along PC${}_{3}$ (about 4% of total variance), the separation of propanoic acid in the negative direction of the scores is found. This is related to CoTPP, which is the only sensor contributing to the negative side of the principal component. Finally, in PC${}_{4}$ (only 1% of total variance) toluene (negative part) and hexane (positive part) are separated from each other. The negative portion of the principal component is due to SnTPPCl2 while the positive portion is contributed by CuTPP, MnTPPCl, and FeTPPCl. In any case, all the scores show a dependence with the concentration which does not allow the full identification of VOCs.

The separation of the qualitative from the quantitative information has been a well known problem since the beginning of the studies on sensor arrays, when a simple method, totally effective in the case of linear sensors, was introduced [23]. This method consists of a normalization of each sensor signal simply dividing the signal of each sensor by the sum of all the others. In the case of an array of N linear sensors the response of the i-th sensor ($\Delta f_{ij}$) to the j-th compound at concentration c ($c_{j}$) is given by:

(1) $$\Delta f_{ij}=S_{ij}\cdot c_{j}$$

where $S_{ij}$ is the sensitivty of the *i*-th sensor to the *j*-th compound. The normalization is achieved by the following operation:

(2) $$\Delta f_{ij}^{*}=\frac{\Delta f_{ij}}{\sum_{k}\Delta f_{kj}}=\frac{S_{ij}c_{j}}{\sum_{k}S_{kj}c_{j}}=\frac{S_{ij}}{\sum_{k}S_{kj}}$$

In practice, the signal of the *i*-th sensor corresponds to its weighted sensitivity.

Obviously, the above equation holds only in case of a linear relationship between the sensor and the concentration. As shown in Figure 2, the response curves are well fitted by a linear function; however, fluctuations, mainly due to imperfections in the measurement setup, exist, so the above introduced normalization typically mitigates the influence of the concentration.

**Figure 6:**
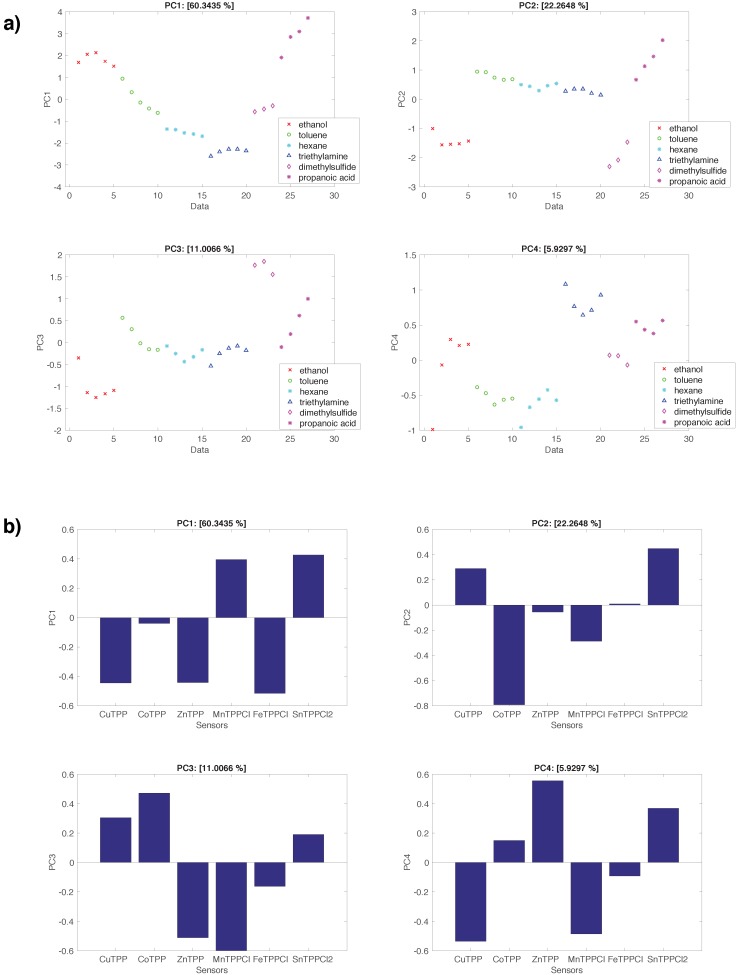
Results of the PCA of the linearly normalized sensors data. (**a**) Scores of the first four principal components; (**b**) Loadings of the first four principal components.

Figure 6 shows the results of the PCA applied to the normalized data. Here, the scores (Figure 6a) show a substantial independence on the concentration and the loadings are well distributed among the scores. Furthermore, each principal component evidences the separation among some of the studied VOCs. PC${}_{1}$ (60% of total variance) are separated between propanoic acid, ethanol (positive direction) and hexane and triethylamine (negative direction). The sensors contributing to the positive score are MnTPPCl and SnTPPCl2, while CuTPP, ZnTPP, and FeTPPCl contributes to the negative part. It is interesting to note that the first principal component of the normalized data is analogue to the second principal component of the non normalized data, which is obtained after the removal of most of the common mode. PC${}_{2}$ (22% of total variance) separates ethanol and dimethylsulfide (negative portion) and propanoic acid (positive portion) and the difference may be attributed to CoTPP and SnTPPCl2, respectively.

Similar considerations can be done for PC${}_{3}$ (11% of total variance) and PC${}_{4}$ (5% of total variance) where the relationship between dimethylsulfide and CuTPP and CoTPP and triethylamine and ZnTPP can be observed.

**Figure 7:**
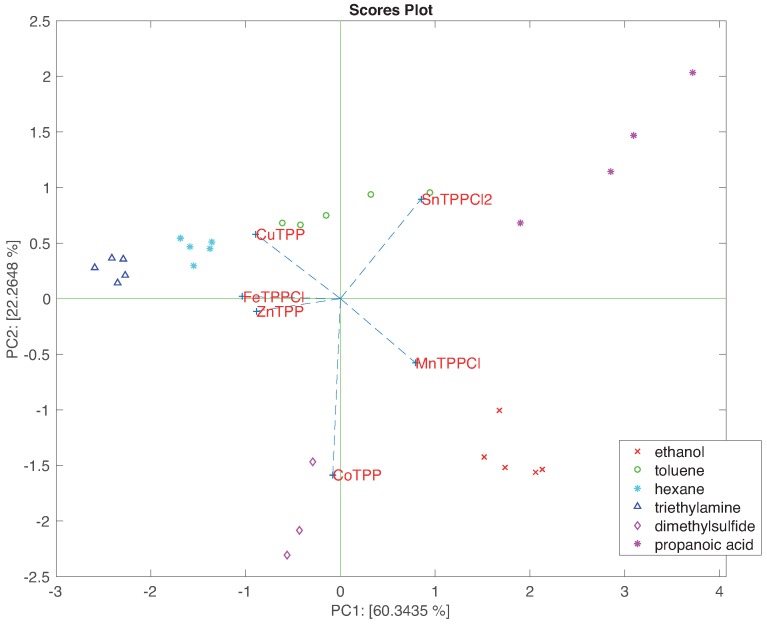
Biplot of the first two principal components of the linearly normalized sensors data.

However, besides analysing the individual principal components, PCA results are typically represented plotting the scores and loadings in planes identified by two principal components. Figure 7 shows the projections of scores and loadings in the plane of the first two principal components. In this plot, the separation among the VOCs is obtained and it is possible to study the correlation between each sensor and scores. In particular, this plot evidences the relationship between propanoic acid and SnTPPCl2, ethanol and MnTPPCl, triethylamine with ZnTPP and FeTPPCl, and finally dimethylsulfide and CoTPP, features that can be confidently correlated to the Pearson theory [24] characteristics of both porphyrin coordinated metal and donor ligand.

## 4. Conclusions

Quartz microbalances are optimal sensors to transduce the whole set of interactions occurring in solid state sensing layer deposited on the sensor surface. In case of metalloporphyrins, the set of interactions spans from the weak Van der Waals forces to hydrogen bond, to π−π interactions and finally to the coordination to the central metal ion. As evidenced by previous studies, all these interactions may contemporaneously be present and cooperate in the total guest molecule binding [25].

In this paper we have investigated the properties of QMBs coated with a homologous series of metalloporphyrins based on the same macrocycle and differing only for the metal atom. Sensors have been tested exposing the devices to different concentrations of six VOCs representative of different chemical families. Results show that all sensors share a common trend, which is largely due to the influence of the macrocycle and the peripheral compounds. On the other hand, superimposed on the common trend, a peculiar behaviour dependent on the metal is clearly evident.

The application of PCA to the sensor array shows that the common modes are mostly captured by the first principal component which accounts for about 60% of the total variance. It is important to consider that, besides the common features of the different metalloporphyrins, an important source of common behaviour is due to the growing concentration of VOCs. Indeed, the response of each sensor is due to the contemporaneous action of the sensitivity, which depends on the nature of the VOC, and the concentration. As a consequence, since each measure of a VOC is taken at different concentration, the identification of VOCs is practically impossible. Most of the common modes might be partially removed by a linear normalization of the sensor array data. It is worth mentioning that the identification of odours regardless of their intensity is a noteworthy property of natural olfaction. The mechanisms of separation of quantitative and qualitative information in olfaction are still largely unknown. This capacity to exclude concentration information does not preclude the olfactory system from estimating concentration itself [26,27].

Results show that PCA applied to normalized data achieves a perfect identification of the VOCs. Furthermore, PCA allows us to study the sensors-VOCs relationship. This relationship fits, in most cases, with a comparison of the sensitivity. As an example, MnTPPCl is the sensor most sensitive to ethanol and it drives the identification of this compound. The interplay of sensors in the array enhances the cooperation among sensors, so even if MnTPPCl shows the largest sensitivity to propanoic acid, this VOCs is actually identified with the cooperation of SnTPPCl2.

These results demonstrate the role of the metal atom in metalloporphyrins based QMB arrays and provide a background for the explanation of the successful applications of these arrays to several real world applications.

## Figures and Tables

**Table 1:** Linear Sorption-Energy Relationship (LSER) parameters of the tested volatile organic compounds (VOCs), R is the polarizability, π> the dipolarity, $\alpha^{H}$ and $\beta^{H}$ the hydrogen bond acidity and basicity, and $LogL^{16}$ is the solubility term [17].

VOC	*R*	*π*	$\alpha^{H}$	$\beta^{H}$	$LogL^{16}$
ethanol	0.246	0.420	0.370	0.480	1.485
toluene	0.601	0.520	0.000	0.140	3.325
hexane	0.000	0.000	0.000	0.000	2.668
triethylamine	0.101	0.150	0.000	0.790	3.040
dimethysulfide	0.404	0.380	0.000	0.290	2.238
propanoic acid	0.233	0.650	0.600	0.450	2.290
